# Efficacy and Safety of Doravirine-based Regimens by Sex and Race: Long-term Results From Three Phase 3 Clinical Trials

**DOI:** 10.1093/ofid/ofaf356

**Published:** 2025-07-16

**Authors:** Sharon L Walmsley, Princy N Kumar, Chloe Orkin, Melanie Thompson, Kathleen Squires, Zhi Jin Xu, Wayne Greaves, Rebeca M Plank, Yohance Whiteside, Rima Lahoulou

**Affiliations:** Department of Medicine, University Health Network, University of Toronto, Toronto, ON, Canada; Division of Infectious Diseases and Tropical Medicine, Georgetown University Medical Center, Washington, DC, USA; Queen Mary University of London, London, UK; Thacker and Thompson, Atlanta, Georgia, USA; MRL Research Laboratories, Merck & Co., Inc., Rahway, New Jersey, USA; MRL Research Laboratories, Merck & Co., Inc., Rahway, New Jersey, USA; MRL Research Laboratories, Merck & Co., Inc., Rahway, New Jersey, USA; MRL Research Laboratories, Merck & Co., Inc., Rahway, New Jersey, USA; MRL Research Laboratories, Merck & Co., Inc., Rahway, New Jersey, USA; MRL Research Laboratories, MSD France, Puteaux, France

**Keywords:** DEI, diversity, human immunodeficiency virus, inclusion, participant selection

## Abstract

**Background:**

Females and persons of Black race are often underrepresented in clinical trials. This post hoc analysis of data from three phase 3 studies evaluated the efficacy and safety of doravirine (DOR) by sex and race in adults living with HIV-1.

**Methods:**

DRIVE-FORWARD and DRIVE-AHEAD open-label extensions were pooled; participants randomized to first-line DOR-based regimen continued from week (W) 96 to W192 (DOR-continued group) and participants randomized to comparators switched to DOR from W96 to W192 (DOR-switch group). In DRIVE-SHIFT, virologically suppressed adults were randomized to switch to a DOR-based regimen on day 1 (immediate-switch group) or W24 (delayed-switch group) and continued through W144. Results are reported by sex assigned at birth (male vs female) and race (Black vs non-Black).

**Results:**

Across trials, female and Black participants each represented <20% of study populations. After continuing or switching to DOR, percentages of participants with HIV-1 RNA <50 copies/mL were comparable between sex and race subgroups. Mean changes in CD4+ T-cell counts and proportions of participants with drug-related adverse events or serious adverse events were generally similar between subgroups. In DRIVE-SHIFT, higher rates of nontreatment-related discontinuations were observed within Black versus non-Black subgroups. Differences in median weight change were generally larger between race subgroups than sex subgroups, although interquartile ranges were wide for all.

**Conclusions:**

Participants who continued or switched to DOR generally had comparable efficacy and safety outcomes across sex and race subgroups. However, the sample size was limited. Future studies should ensure greater diversity when investigating factors leading to outcome disparities.

**ClinicalTrials.gov:** NCT02275780, NCT02403674, NCT02397096.

## Teaser

This post hoc analysis evaluated the efficacy and safety of doravirine-based regimens across sex and race subgroups in three phase 3 clinical trials. Outcomes were comparable between subgroups, but greater diversity in future trials is encouraged to assess potential outcome disparities.

Demographic parameters, including sex assigned at birth, gender identification, age, race, or ethnicity, may have effects on treatment efficacy and safety that cannot be predicted when evaluating a new investigational drug [1–5]. To ensure equitable access to health advances and the generalizability of research findings, many investigators, agencies, and advocacy groups have prioritized improved inclusivity of diverse populations in clinical trials to help identify any potential effects of demographic parameters on efficacy or tolerability early in the clinical development process [6–9].

Females and persons of Black race constitute the majority of those living with HIV worldwide; however, the response to the global HIV epidemic has historically been delayed and disparate in these populations, in which these individuals remain underrepresented in HIV antiretroviral clinical trials [1, 2]. As a consequence, pooling data from clinical trials has been necessary to evaluate sufficient numbers in these demographics. For example, 2 analyses that used pooled data from phase 3 clinical trials of integrase inhibitor-based regimens in participants living with HIV-1 (first-line and treatment experienced) demonstrated that response to antiretroviral therapy can vary by sex and race [1, 2]. The proportion of participants who achieved virologic suppression was numerically lower among participants who were Black (vs non-Black) [2], female (vs male) [2], and Black male (vs Black female or non-Black male) [1]. The differences observed in these trials may be due to higher early discontinuation rates in these subgroups [1, 2]. These reports highlight that findings from clinical trials lacking in participant diversity may not be generalizable to all people living with HIV, as effectiveness and tolerability may not have been assessed for sex and race subpopulations, and emphasize the importance of accurately representing the demographics of HIV populations globally in clinical trial enrollment.

Doravirine (DOR) is a nonnucleoside reverse transcriptase inhibitor approved for use in combination with other antiretroviral agents for the treatment of HIV-1 in adults and adolescents [10, 11]. In 2 double-blind randomized phase 3 clinical trials of first-line therapy in adults living with HIV-1 (DRIVE-FORWARD [NCT02275780] and DRIVE-AHEAD [NCT02403674]), DOR-based regimens demonstrated noninferior efficacy and a favorable safety profile through 48 and 96 weeks compared with ritonavir-boosted darunavir- and efavirenz-based regimens, respectively [12–16]. Furthermore, in the open-label extensions of these trials, the favorable efficacy and safety seen with DOR were maintained through to week 192 in participants who continued or switched to DOR-based regimens [17]. In the phase 3 clinical trial of virologically suppressed adults living with HIV-1 (DRIVE-SHIFT; NCT02397096), switching to a DOR-based regimen was noninferior to continuing the baseline regimen for maintaining viral suppression, and the DOR-based regimen was generally well tolerated through 48 and 144 weeks [18, 19]. The current post hoc analysis was conducted to evaluate the efficacy and safety of DOR by sex assigned at birth and by race using combined data from these three phase 3 clinical trials.

## METHODS

### Study Design

Data from the DRIVE-FORWARD (MK-1439-018), DRIVE-AHEAD (MK-1439A-021) (Supplementary Figure 1A), and DRIVE-SHIFT (MK-1439A-024) (Supplementary Figure 1B) trials were used in this post hoc analysis. In DRIVE-FORWARD, adults living with HIV-1 received first-line treatment with either DOR or ritonavir-boosted darunavir, and an investigator-selected nucleoside reverse transcriptase inhibitor regimen of either tenofovir disoproxil fumarate (TDF) and emtricitabine (FTC), or abacavir and lamivudine (3TC), with all participants receiving a DOR-based regimen in the open-label extension (weeks 96–192). In DRIVE-AHEAD, adults living with HIV-1 received first-line treatment with a daily fixed-dose tablet of either DOR/3TC/TDF (100/300/300 mg) or efavirenz/FTC/TDF (600/200/300 mg), with all participants receiving DOR/3TC/TDF in the open-label extension (weeks 96–192). In DRIVE-SHIFT, treatment-experienced adults living with HIV-1 on a stable regimen (ritonavir- or cobicistat-boosted protease inhibitor, cobicistat-boosted elvitegravir, or a nonnucleoside reverse transcriptase inhibitor, each in combination with 2 nucleoside reverse transcriptase inhibitors) were switched to a daily fixed-dose tablet of DOR/3TC/TDF (100/300/300 mg) on either day 1 or week 24 and continued to receive DOR/3TC/TDF through week 144. Results of these studies have been previously published, with the methods described in detail [17, 19].

This post hoc analysis included a DOR-continued group of participants from DRIVE-FORWARD and DRIVE-AHEAD (pooled data) who were previously untreated and initially randomly assigned to a first-line DOR-based regimen and continued these regimens in the open-label extension phases (weeks 96–192). The DOR-switch group included participants from DRIVE-FORWARD and DRIVE-AHEAD (pooled data) who were initially randomly assigned to a first-line comparator regimen and then switched to the DOR-based regimen at week 96 through the open-label extension phases (weeks 96–192). Only trial data from the open-label extension phases (weeks 96–192) of DRIVE-FORWARD and DRIVE-AHEAD were included in this analysis.

This post hoc analysis also included an immediate-switch group consisting of participants from DRIVE-SHIFT, who were virologically suppressed and randomly assigned to switch to a DOR-based regimen on day 1 and continued this regimen through week 144. The delayed-switch group included participants from DRIVE-SHIFT who were randomly assigned to switch to a DOR-based regimen from week 24 through week 144. This analysis included trial data from the base study and extension phase of DRIVE-SHIFT (day 1 to week 144) for the participants who entered the extension phase (weeks 48–144) only.

### Statistical Analysis

Efficacy endpoints were the proportion of participants with HIV-1 RNA <50 copies/mL using the US Food and Drug Administration (FDA) snapshot and observed failure approaches, and the mean change from baseline in CD4+ T-cell counts at week 192 (DRIVE-FORWARD and DRIVE-AHEAD) and Week 144 (DRIVE-SHIFT). For DRIVE-FORWARD and DRIVE-AHEAD, the mean change from baseline for CD4+ T-cell counts was calculated from weeks 96 to 192. For DRIVE-SHIFT, the mean change from baseline for CD4+ T-cell counts was calculated from day 1 to week 144 for the immediate-switch group and from weeks 24 to 144 for the delayed-switch group. For the FDA snapshot approach, all missing data were treated as failures, regardless of the reason, and for the observed failure approach, baseline values were carried forward for participants who discontinued assigned therapy due to lack of efficacy and other missing values were excluded from analysis.

At each study visit, safety was monitored by adverse events (AEs) reporting. AEs were assessed by the investigator for intensity (mild, moderate, severe), seriousness, and relationship to study therapy.

In addition to the efficacy and safety analyses, the median change in weight was calculated. For DRIVE-FORWARD and DRIVE-AHEAD, median weight change was calculated from weeks 96 to 192. For DRIVE-SHIFT, median weight change was calculated from day 1 to week 144 for the immediate-switch group and from weeks 24 to 144 for the delayed-switch group.

All results are reported by sex assigned at birth (male vs female) and race (Black vs non-Black). Races included in the non-Black category were American Indian/Alaska Native, Asian, Hawaiian/other Pacific Islander, and White. Data from participants reporting multiple races were treated as missing in the Black versus non-Black comparisons. Median change in weight was also reported in race/sex subgroups of Black female, Black male, non-Black female, and non-Black male. No adjustment was made for multiple comparisons, and this post hoc analysis was not designed to detect statistically significant differences within or between subgroups.

## RESULTS

### Study Population

Of the 1494 participants treated in the double-blind phases of the DRIVE-FORWARD and DRIVE-AHEAD trials, 550 DOR-continued and 502 DOR-switch participants entered the open-label extensions (weeks 96–192) [17]. Furthermore, 656 participants were switched to DOR in the DRIVE-SHIFT trial and 600 entered the extension to week 144 [19], including 398 immediate-switch and 202 delayed-switch participants. Accordingly, a total of 1652 participants were included in this post hoc analysis (Table 1). Female participants and Black participants each represented <20% of the total study populations in each of the 3 trials, where <8% were in the Black female subgroup (Table 1).

**Table 1.** Participant Demographics

Participants	DRIVE-FORWARD/DRIVE-AHEAD		DRIVE-SHIFT	
	DOR Continued N = 550	DOR Switch N = 502	Immediate Switch^a^ N = 398	Delayed Switch^a^ N = 202
Age, years				
Mean (SD)	34.6 (10.5)	34.0 (10.4)	43.4 (10.0)	43.8 (10.4)
Median (range)	33.0 (18–70)	32.0 (18–69)	44.0 (21–71)	42.5 (22–71)
Sex, n (%)				
Female	93 (16.9)	68 (13.5)	68 (17.1)	27 (13.4)
Male	457 (83.1)	434 (86.5)	330 (82.9)	175 (86.6)
Race^b^, n (%)				
Black	100 (18.2)	86 (17.1)	50 (12.6)	28 (13.9)
Non-Black	405 (73.6)	371 (73.9)	325 (81.7)	163 (80.7)
American Indian/Alaska Native	7 (1.3)	6 (1.2)	5 (1.3)	2 (1.0)
Asian	58 (10.5)	53 (10.6)	15 (3.8)	6 (3.0)
Native Hawaiian/other Pacific Islander	0	2 (0.4)	1 (0.3)	0
White	340 (61.8)	310 (61.8)	304 (76.4)	155 (76.7)
Missing	45 (8.2)	45 (9.0)	23 (5.8)	11 (5.4)
Race/sex				
Black female	42 (7.6)	29 (5.8)	24 (6.0)	13 (6.4)
Non-Black female	45 (8.2)	36 (7.2)	42 (10.6)	13 (6.4)
Black male	58 (10.5)	57 (11.4)	26 (6.5)	15 (7.4)
Non-Black male	360 (65.5)	335 (66.7)	283 (71.1)	150 (74.3)
Missing	45 (8.2)	45 (9.0)	23 (5.8)	11 (5.4)

Abbreviation: DOR, doravirine.

^a^Only participants who entered the DRIVE-SHIFT extension phase from weeks 48 to 144 were included in the analyses for the immediate-switch and delayed-switch groups.

^b^Races included in the non-Black category were American Indian/Alaska Native, Asian, Hawaiian/other Pacific Islander, and White.

### Efficacy

**Figure 1.**
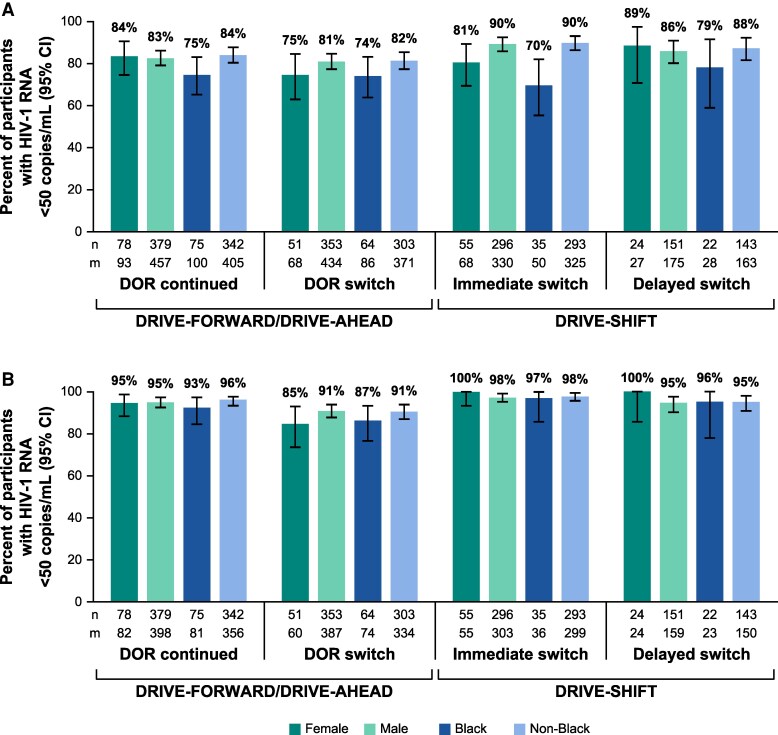
Proportion of participants with HIV-1 RNA <50 copies/mL by sex and race^a^ at week 192 (DRIVE-FORWARD/DRIVE-AHEAD) and week 144 (DRIVE-SHIFT^b^) per the (*A*) FDA snapshot approach and (*B*) observed failure approach. DOR, doravirine; FDA, US Food and Drug Administration; m, number of participants in each subgroup; n, number of participants in each subcategory. ^a^Multiple/missing races not included in the Black or non-Black groups: 45 each for the DOR-continued and the DOR-switch groups; 23 for the immediate-switch group; 11 for the delayed-switch group. ^b^Races included in the non-Black category were American Indian/Alaska Native, Asian, Hawaiian/other Pacific Islander, and White.

After continuing or switching to a DOR-based regimen in DRIVE-FORWARD, DRIVE-AHEAD, and DRIVE-SHIFT, the percentage of participants with virologic suppression (HIV-1 RNA <50 copies/mL) was generally comparable between the male and female subgroups and between Black and non-Black participants (Figure 1). When analyzed using the FDA snapshot approach, Black participants in the immediate-switch group in DRIVE-SHIFT exhibited a lower rate of virologic suppression (70.0%; 95% confidence interval [CI], 55.4–82.1) compared with non-Black participants (90.2% [86.4–93.2]), noting that the proportion of participants with no virologic data available at the time of evaluation was higher among the Black subgroup than the non-Black subgroup in DRIVE-SHIFT (28% vs 8.0%, respectively). This was primarily driven by a substantially higher rate of discontinuation for non–treatment-related reasons within the Black subgroup compared with the non-Black subgroup (28% vs 4.6%, respectively). The proportion of participants with no virologic data was generally comparable between the Black and non-Black subgroups in DRIVE-AHEAD (19% vs 12%, respectively) and DRIVE-FORWARD (14% vs 10%, respectively). This difference in rate of virologic suppression between race subgroups was not seen when applying the observed failure approach (Black participants: 97.2% [85.5–99.9]; non-Black participants (98.0% [95.7–99.3]).

**Figure 2.**
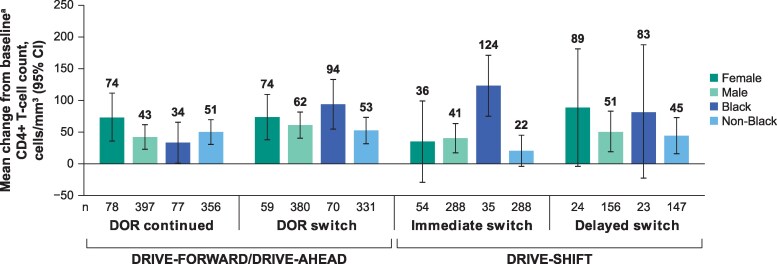
Mean change from baseline^a^ in CD4+ T-cell counts by sex and race^b,c^ at week 192 (DRIVE-FORWARD/DRIVE-AHEAD) and week 144 (DRIVE-SHIFT). DOR, doravirine; n, number of participants in each subcategory. ^a^Mean change from baseline CD4+ T-cell counts was analyzed from weeks 96 to 192 for the DOR-continued group and the DOR-switch group, from day 1 to week 144 for the immediate-switch group, and from weeks 24 to 144 for the delayed-switch group. ^b^Multiple/missing races not included in the Black or non-Black groups: 45 each for the DOR-continued and the DOR-switch groups; 23 for the immediate-switch group; 11 for the delayed-switch group. ^c^Races included in the non-Black category were American Indian/Alaska Native, Asian, Hawaiian/other Pacific Islander, and White.

Mean change in CD4+ T-cell counts was generally comparable between the male and female subgroups and between Black and non-Black participants, except in the immediate-switch group in DRIVE-SHIFT (Figure 2). A greater increase in CD4+ T-cell count was observed in Black compared with non-Black participants in the immediate-switch group (124 cells/mm^3^ [76.1–172.3 cells/mm^3^] vs 22 cells/mm^3^ [−2.9 to 46.0 cells/mm^3^]); baseline CD4 cell counts in the Black and non-Black subgroups were 599 and 684 cells/mm^3^, respectively.

### Adverse Events

In the DOR-continued and the DOR-switch groups (DRIVE-FORWARD/DRIVE-AHEAD), the proportion of participants who experienced drug–related AEs was higher among male subgroups (DOR-continued: 8.3%; DOR–switch: 8.1%) than female subgroups (DOR-continued: 0%; DOR-switch: 2.9%) and was similar between the Black (DOR-continued: 6.0%; DOR-switch: 5.8%) and the non-Black subgroups (DOR-continued: 6.9%; DOR-switch: 7.8%; Table 2). In DRIVE-SHIFT, the proportion of participants who experienced drug-related AEs was higher in female (immediate-switch: 29.4%; delayed-switch: 29.6%) compared with male subgroups (immediate-switch: 23.0%; delayed-switch: 14.3%). There was no consistent trend in drug-related AEs across the race subgroups in the immediate switch (Black: 18.0%; non-Black: 24.9%) and delayed-switch arms (Black: 25.0%; non-Black: 15.3%) in DRIVE-SHIFT. Across trials and subgroups, discontinuation resulting from drug-related AEs was uncommon; none was reported among females or Black subgroups (Table 2).

**Table 2.** Proportion of Participants Who Experienced AEs by Sex and Race^a^ at Week 192 (DRIVE-FORWARD/DRIVE-AHEAD) and Week 144 (DRIVE-SHIFT)

	DRIVE-FORWARD/DRIVE-AHEAD				DRIVE-SHIFT			
	DOR Continued N = 550		DOR Switch N = 502		Immediate Switch N = 398		Delayed Switch N = 202	
	Female n = 93	Male n = 457	Female n = 68	Male n = 432	Female n = 68	Male n = 330	Female n = 27	Male n = 175
≥1 AE	71 (76.3)	338 (74.0)	46 (67.6)	332 (76.5)	62 (91.2)	301 (91.2)	23 (85.2)	149 (85.1)
Discontinued due to any AE	0	6 (1.3)	0	4 (0.9)	0	7 (2.1)	0	5 (2.9)
≥1 drug-related AE	0	38 (8.3)	2 (2.9)	35 (8.1)	20 (29.4)	76 (23.0)	8 (29.6)	25 (14.3)
Discontinued due to drug-related AE	0	3 (0.7)	0	1 (0.2)	0	4 (1.2)	0	4 (2.3)
≥1 serious AE	8 (8.6)	28 (6.1)	3 (4.4)	25 (5.8)	8 (11.8)	38 (11.5)	3 (11.1)	12 (6.9)
	Black n = 100	Non-Black^b^ n = 405	Black n = 85	Non-Black^b^ n = 370	Black n = 50	Non-Black^b^ n = 325	Black n = 28	Non-Black^b^ n = 163
≥1 AE	75 (75.0)	296 (73.1)	61 (70.9)	283 (76.3)	45 (90.0)	297 (91.4)	22 (78.6)	142 (87.1)
Discontinued due to any AE	0	6 (1.5)	0	2 (0.5)	0	7 (2.2)	0	5 (3.1)
≥1 drug-related AE	6 (6.0)	28 (6.9)	5 (5.8)	29 (7.8)	9 (18.0)	81 (24.9)	7 (25.0)	25 (15.3)
Discontinued due to drug-related AE	0	3 (0.7)	0	0	0	4 (1.2)	0	4 (2.5)
≥1 serious AE	5 (5.0)	30 (7.4)	5 (5.8)	22 (5.9)	5 (10.0)	37 (11.4)	1 (3.6)	14 (8.6)

Abbreviations: AE, adverse event; DOR, doravirine.

^a^Multiple/missing races not included in the Black or non-Black groups: 45 each for the DOR-continued and the DOR-switch groups; 23 for the immediate-switch group; 11 for the delayed-switch group.

^b^Races included in the non-Black category were American Indian/Alaska Native, Asian, Hawaiian/other Pacific Islander, and White.

Serious AEs were similar between female (DOR-continued: 8.6%; DOR-switch: 4.4%) and male (DOR-continued: 6.1%; DOR-switch: 5.8%) subgroups in DRIVE–FORWARD/DRIVE-AHEAD, and in the immediate-switch group from DRIVE-SHIFT (11.8%, and 11.5%, respectively) but were higher among female compared with male participants in the delayed-switch group from DRIVE-SHIFT (11.1%, and 6.9%, respectively; Table 2).

### Weight Change

Across the 3 trials, differences in median weight change were generally larger between race subgroups (Black vs non-Black) than those between the sex subgroups (female vs male), although interquartile ranges were wide for all (Table 3). In the DRIVE-FORWARD and DRIVE-AHEAD trials, median weight gain was greater among Black participants (3.6 kg) than non-Black participants (1.6 kg) in the DOR-switch group (Table 3), with similar weight gain observed in the Black female (4.4 kg) and Black male subgroups (3.4 kg; Supplementary Figure 2). Median weight gain was also higher among Black participants (2.5 kg) than non-Black participants (1.0 kg) in the delayed-switch group in DRIVE-SHIFT (Table 3), with higher weight gain observed in the Black female subgroup (4.5 kg) than in the Black male subgroup (0.8 kg) (Supplementary Figure 2).

**Table 3.** Median Weight Change by Sex and Race^a^ at Week 192 (DRIVE-FORWARD/DRIVE-AHEAD) and Week 144 (DRIVE SHIFT)

	DRIVE-FORWARD/DRIVE-AHEAD				DRIVE-SHIFT			
	DOR Continued N = 550		DOR Switch N = 502		Immediate Switch N = 398		Delayed Switch N = 202	
	Female n = 93	Male n = 457	Female n = 68	Male n = 432	Female n = 68	Male n = 330	Female n = 27	Male n = 175
Weight change,^b^ median (IQR), kg	0.1 (−2.6 to 4.7)	0.7 (−1.5 to 3.4)	1.9 (−0.5 to 5.2)	1.7 (−0.9 to 4.5)	0.0 (−2.8 to 2.6)	1.0 (−1.4 to 4.5)	2.0 (−1.1 to 3.7)	1.0 (−1.0 to 4.1)
	Black n = 100	Non-Black^c^ n = 405	Black n = 85	Non-Black^c^ n = 370	Black n = 50	Non-Black^c^ n = 325	Black n = 28	Non-Black^c^ n = 163
Weight change,^b^ median (IQR), kg	0.9 (−1.6 to 4.9)	0.7 (−1.6 to 3.5)	3.6 (0.1 to 7.2)	1.6 (−1.0 to 4.3)	1.0 (−1.7 to 4.0)	0.9 (−1.8 to 4.0)	2.5 (−0.9 to 5.7)	1.0 (−1.3 to 3.9)

Abbreviations: DOR, doravirine; IQR, interquartile range.

^a^Multiple/missing races not included in the Black or non-Black groups: 45 each for the DOR-continued and the DOR-switch groups; 23 for the immediate-switch group; 11 for the delayed-switch group.

^b^Median weight change was analyzed from weeks 96 to 192 for the DOR-continued and the DOR-switch groups, from day 1 to week 144 for the immediate-switch group, and from weeks 24 to 144 for the delayed-switch group.

^c^Races included in the non-Black category were American Indian/Alaska Native, Asian, Hawaiian/other Pacific Islander, and White.

## DISCUSSION

After continuing or switching to DOR-based regimens in the phase 3 DRIVE-FORWARD, DRIVE-AHEAD, and DRIVE-SHIFT clinical trials, participants generally had comparable long–term efficacy outcomes across sex (male vs female) and race (Black vs non-Black) subgroups. Rates of virological suppression were lower among Black participants in the immediate-switch group in DRIVE-SHIFT when analyzed using the FDA snapshot approach, consistent with some, but not all, previous clinical studies of integrase inhibitor-based regimens in participants living with HIV-1 [1, 2]. However, this difference was not apparent when applying the observed failure approach. Considering the substantially higher rate of discontinuation for non–treatment-related reasons within the Black subgroup compared with the non-Black subgroup, this highlights that missing data can have a substantial effect when using the FDA snapshot approach and can impact the analysis of long-term follow-up data. Furthermore, other endpoints, such as early discontinuation for non–treatment-related reasons, warrant further investigation, particularly as such outcomes may be more common in certain subgroups. Although the underlying reasons for the disparity in discontinuation due to non–treatment-related reasons between race subgroups were not explored in these studies, these findings motivate further investigation into social determinants of health and societal inequities affecting different demographic subpopulations.

The greater increase in CD4+ T-cell count observed in Black compared with non-Black participants in the immediate-switch group of DRIVE-SHIFT was possibly related to the comparatively lower baseline mean CD4+ T-cell count observed in the Black subgroup. However, the observed differences were small and not likely to be clinically meaningful.

The proportions of participants who experienced drug-related AEs differed according to sex (male vs female) and race (Black vs non-Black) within the various study arms, but these subgroup differences were not directionally consistent across arms. However, the incidence of AEs differed across the 3 trials. The higher percentage of AEs and drug-related AEs observed in the DRIVE-SHIFT study compared with the DRIVE-FORWARD and DRIVE-AHEAD studies may be related to the different reporting time frames used in the current analysis to evaluate AEs in the 3 trials. In DRIVE–FORWARD and DRIVE-AHEAD, AEs reported from week 96 to week 192 were analyzed; in DRIVE–SHIFT, AEs reported from the time participants were enrolled in the trial were analyzed (day 1 for immediate-switch group, week 24 for delayed-switch group). Because AEs may occur more frequently in the earlier stages of clinical trials, this difference in reporting time frame may account, in part, for the higher incidence of AEs in DRIVE-SHIFT. Additionally, AEs and drug-related AEs observed in these trials may be related to any agent used in the various DOR-based regimens included in these analyses (DOR with FTC/TDF or abacavir/3TC in DRIVE-FORWARD; DOR/3TC/TDF in DRIVE-AHEAD and DRIVE-SHIFT).

Weight gain was greatest in the Black female subgroup in the DOR-switch arm of DRIVE-FORWARD/DRIVE-AHEAD (median [interquartile range]: 4.4 [0.9–9.1] kg), and in the delayed-switch arm of DRIVE-SHIFT (median [interquartile range]: 4.5 [−6.3 to 7.3] kg), consistent with reports for some other antiretroviral agents [20]. However, the small number of Black female participants enrolled in these studies limited a robust analysis. A previous pooled analysis of double-blind data through week 96 from the first-line DRIVE-AHEAD and DRIVE-FORWARD studies and a phase 2b study of DOR reported similar outcomes; although sex assigned at birth (female or male) and race (Black or non-Black) were not significantly associated with ≥10% weight gain or a body mass index class increase, mean weight gain was numerically higher in the female (vs male) and Black (vs non-Black) subgroups throughout the various analyses [21]. As for the AE results, the weight gain outcomes observed in these trials may be confounded by the agents used in the different DOR-based regimens evaluated in these analyses, as certain agents, such as TDF, have previously shown weight attenuation effects while others have been associated with weight gain [22]. However, the weight gain observed with DOR-based regimens in the DRIVE-FORWARD and DRIVE-AHEAD studies overall was minimal: the median weight gain over up to 4 years of treatment was approximately 2 kg in both trials [17], similar to the average yearly increase observed among US adults without HIV [23]. Any influence of prior regimen was not evaluated in this study.

Although the observations according to sex and race subgroup presented here were consistent with the overall findings from the three phase 3 studies, this post hoc analysis was limited by a small sample size and lack of power to detect statistically significant differences within or between these subgroups, possibly impacting the interpretation of the results. Another limitation of this study was the inclusion of data only from participants who entered the open-label extension phases of the 3 trials. This may have led to the inclusion of individuals who achieved better treatment and safety outcomes, potentially masking additional race and sex differences. Because of the small number of participants, data from those who identified as American Indian/Alaska Native, Asian, and Hawaiian/other Pacific Islander were pooled with White participants in the non-Black group for this analysis. Another limitation of this analysis was that sex assigned at birth was collected in these trials, but gender identification was not. To allow for more in-depth and robust analyses of outcomes across population subgroups and to ensure generalizability of research findings, future studies should prioritize enrollment diversity with regard to sex, gender, ethnic, and racial identifications. Additionally, further research endeavors should identify and address health and societal inequities faced by female and Black participants to improve study retention by minimizing discontinuation for non–treatment-related reasons and to ensure equitable access to health advances. This support will continue to play a key role in translating clinical trial results into real-world practice.

## CONCLUSIONS

Long-term efficacy outcomes and safety profiles were generally comparable regardless of sex assigned at birth (male vs female) and race (Black vs non-Black) among participants continuing or switching to DOR-based regimens in the DRIVE-FORWARD, DRIVE-AHEAD, and DRIVE-SHIFT trials. However, the small sample size may limit the generalizability of the findings. A lower rate of virologic suppression was observed in Black participants in the immediate-switch group in DRIVE-SHIFT most likely due to a high rate of discontinuations in this subgroup. Ensuring optimal representation for greater diversity in future studies, including gender, race, and affected groups, is crucial to understanding and addressing outcome disparities through targeted support and improved retention.

## Supplementary Material

ofaf356_Supplementary_Data

## Supplementary Data

Supplementary materials are available at *Open Forum Infectious Diseases* online. Consisting of data provided by the authors to benefit the reader, the posted materials are not copyedited and are the sole responsibility of the authors, so questions or comments should be addressed to the corresponding author.

## Notes

***Acknowledgments.*** The authors thank all the individuals who participated in these phase 3 studies (DRIVE-FORWARD, DRIVE-AHEAD, and DRIVE-SHIFT). The contributions of the investigators and their staff are also gratefully recognized. Medical writing and/or editorial assistance was provided by Kate Sendin, PhD, and Claire Pouwels of ApotheCom (London, UK). This assistance was funded by Merck Sharp & Dohme LLC, a subsidiary of Merck & Co., Inc., Rahway, NJ, USA.

***Author Contributions.*** S.L.W., P.N.K., C.O., M.T., K.S., Z.J.X., W.G., R.M.P., Y.W., and R.L. contributed to the conception, design, or planning of the study; and/or the acquisition or analysis of the data and/or interpretation of the results; and substantially contributed to the drafting of the manuscript and/or critically reviewed or revised it for important intellectual content. All authors provided final approval of the version to be published and are responsible for the work described in this article.

***Data sharing.*** The data sharing policy, including restrictions, of Merck Sharp & Dohme LLC, a subsidiary of Merck & Co., Inc., Rahway, NJ, USA (MSD) is available at: https://trialstransparency.msdclinicaltrials.com/policies-perspectives.aspx. Requests for access to the clinical study data can be submitted via email to the Data Access mailbox (mailto:dataaccess@msd.com).

***Patient consent statement.*** DRIVE-FORWARD, DRIVE-AHEAD, and DRIVE-SHIFT trials were conducted in conformance with International Council for Harmonisation Guidelines for Good Clinical Practice and applicable regulations regarding the protection of human participants in biomedical research. The protocols were approved by the independent ethics committee for each study site, and all participants provided written informed consent before any study procedures were performed. No additional data were collected for the current post hoc analysis.

***Financial support.*** Funding for this research was provided by Merck Sharp & Dohme LLC, a subsidiary of Merck & Co., Inc., Rahway, NJ, USA.
