# Supplementary material for: Efficacy and Safety of Doravirine-based Regimens by Sex and Race: Long-term Results From Three Phase 3 Clinical Trials
Source: Open Forum Infect Dis. 2025 Jul 16;12(7):ofaf356. doi: 10.1093/ofid/ofaf356 (PMC12264332; doi:10.1093/ofid/ofaf356)
Supplement: ofaf356_Supplementary_Data [file ofaf356_supplementary_data.docx]

**Supplementary Figure 1.** Study designs of (A) DRIVE-FORWARD and DRIVE-AHEAD and (B) DRIVE-SHIFT.

**A**
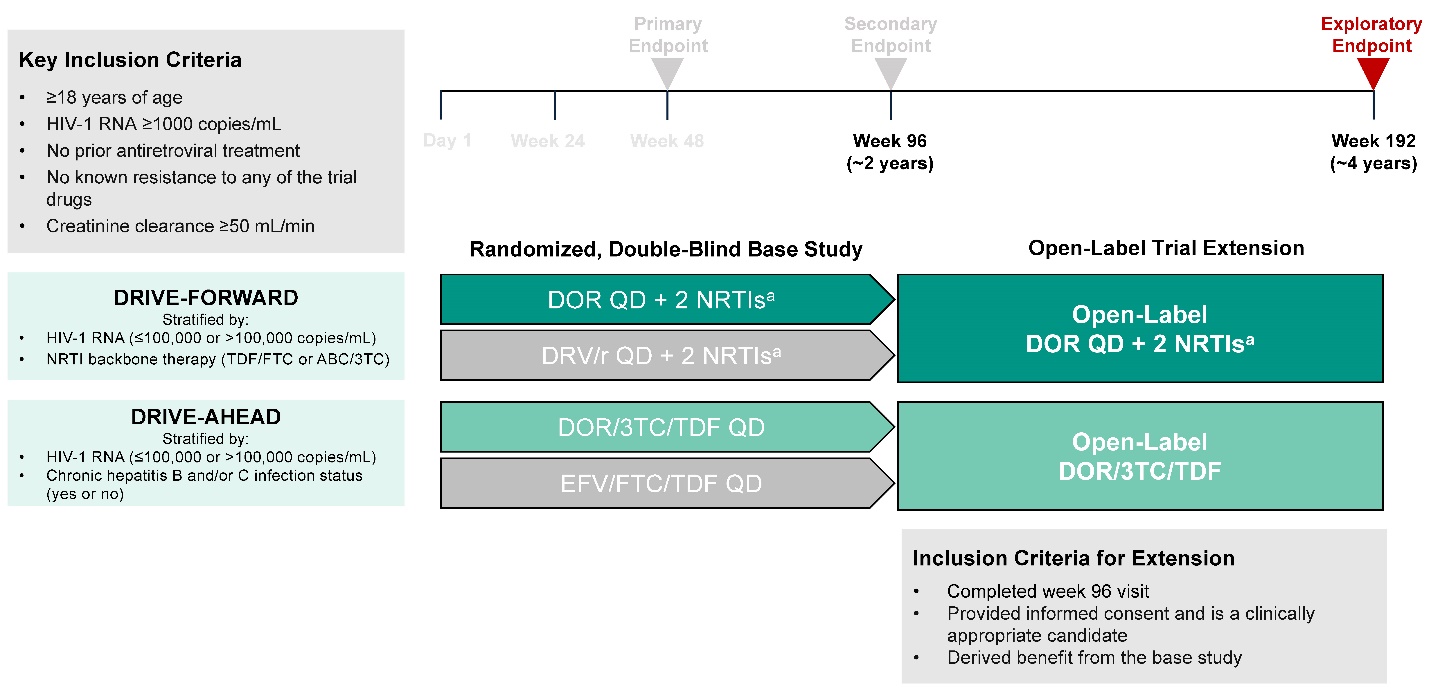
**B**
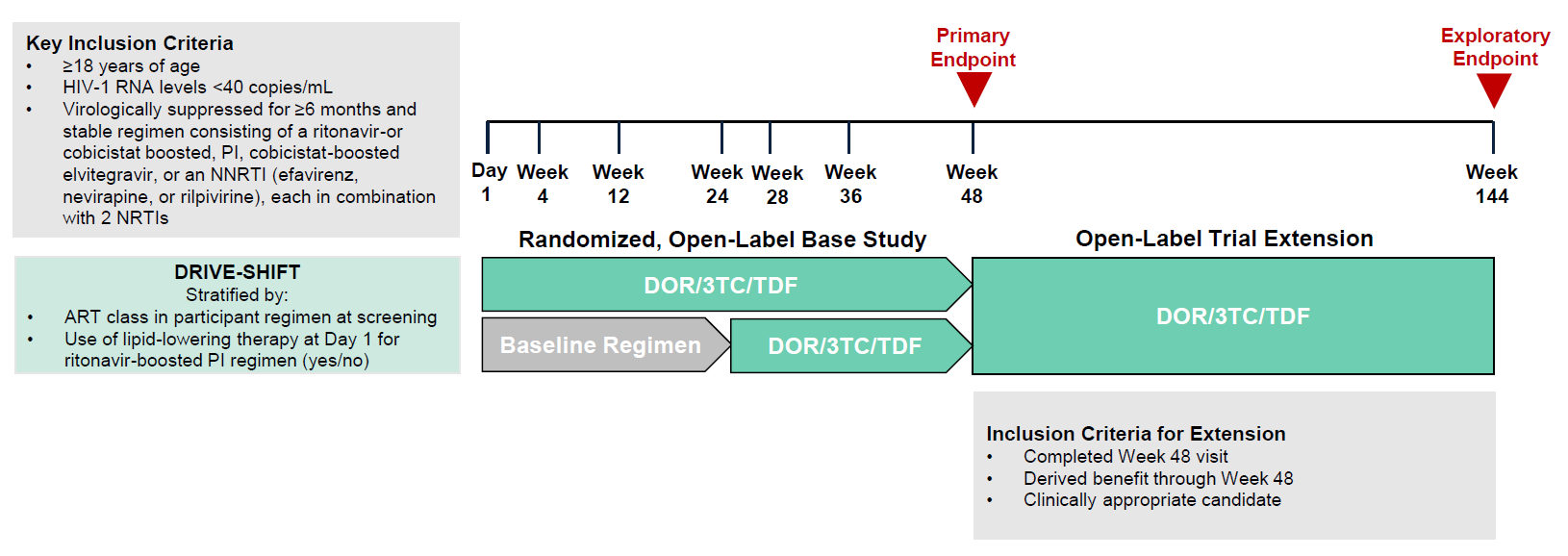


3TC, lamivudine; ABC, abacavir; ART, antiretroviral therapy; DOR, doravirine; DRV/r, ritonavir-boosted darunavir; EFV, efavirenz; FTC, emtricitabine; NNRTI, non-nucleoside reverse transcriptase inhibitor; NRTI, nucleos(t)ide reverse transcriptase inhibitor; PI, protease inhibitor; QD, once daily, TDF, tenofovir disoproxil fumarate.
^a^NRTIs were TDF/FTC or ABC/3TC.

**Supplementary Figure 2.** Median weight change^a^ by sex/race^b^ at Week 192 (DRIVE-FORWARD/DRIVE-AHEAD) and Week 144 (DRIVE-SHIFT).


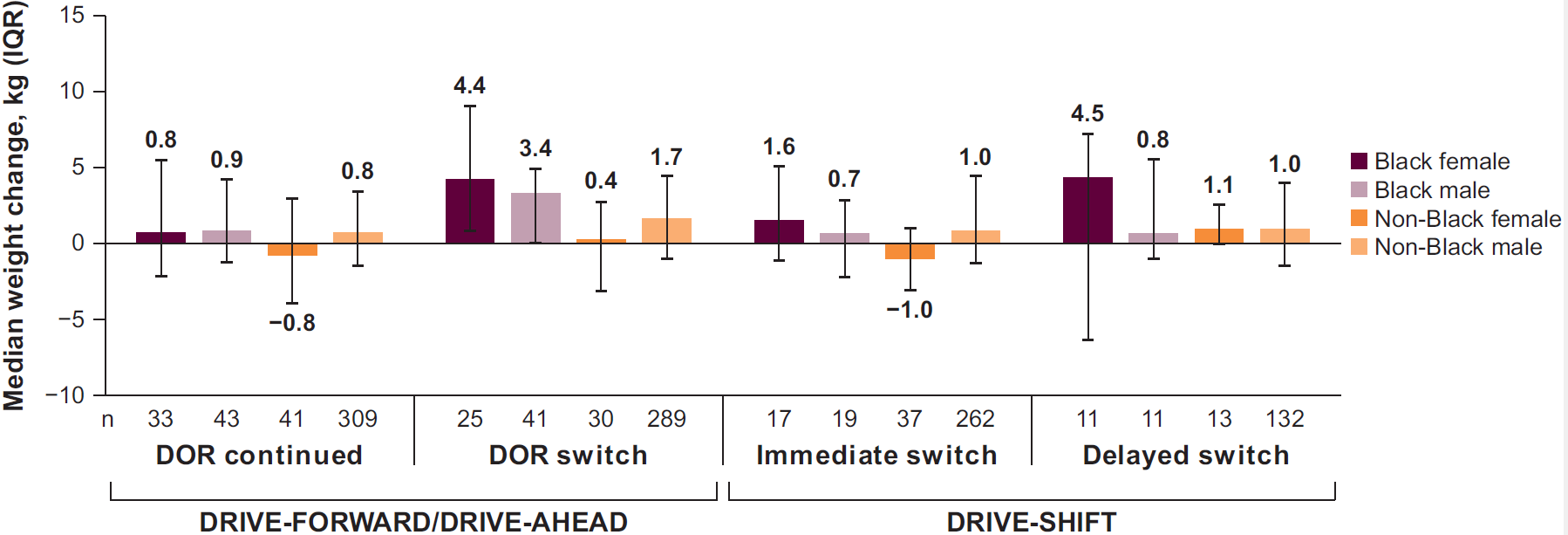


DOR, doravirine; IQR, interquartile range; n, number of participants in each subcategory.

^a^Median weight change (IQR) was analyzed from Weeks 96 to 192 for the DOR-continued and the DOR-switch groups, from Day 1 to Week 144 for the immediate-switch group, and from Weeks 24 to 144 for delayed-switch group.

^b^Races included in the non-Black category were American Indian/Alaska Native, Asian, Hawaiian/other Pacific Islander, and White.
